# Estimation of the Deformation Gradient Tensor by Particle Tracking Near a Free Boundary with Quantified Error

**DOI:** 10.1007/s11340-023-00981-8

**Published:** 2023-08-11

**Authors:** T. Benkley, C. Li, J. Kolinski

**Affiliations:** School of Engineering, École Polytechnique Fédérale de Lausanne, Lausanne, 1015 Switzerland

**Keywords:** Deformation gradient tensor, Particle tracking, Error quantification, Fracture mechanics

## Abstract

**Background:**

Obtaining accurate displacement measurements for large material deformation and/or rotation presents a distinct challenge to digital image correlation (DIC) due to cumulative and decorrelation errors, particularly near material boundaries.

**Objective:**

We aim to accurately measure the deformation gradient tensor near boundary discontinuities in situations of large deformation and large deformation gradients.

**Methods:**

To achieve this goal, the locations of randomly distributed particles are tracked using an open-source particle-tracking software, Trackpy. A least-squares estimate of the deformation gradient tensor field uses nearest-neighbor material vectors and a first-order Finite Difference (FD) approximation, circumventing common errors in other methods. The error caused by FD approximation and that incurred by measurement are derived and tested with exhaustive numerical simulations. Furthermore, a uniaxial tensile test and mode-I fracture experiment are conducted with particle-embedded hydrogels to validate the method.

**Results:**

Numerical results corroborate a theoretical expression of measurement error. They show that the FD error increases while the measurement error decreases for a growing estimating radius. Moreover, measurement error is linearly correlated to displacement noise. A benchmark uniaxial tensile test validates the accuracy of the proposed estimator, and the near-crack-tip measurements in a tensile fracture experiment demonstrate the estimator’s capabilities near a free surface, when a material undergoes large deformation and rotation. The results of the displacement and strain data are benchmarked against kinematic data obtained using an open-source DIC software, Ncorr. Computation time for both methods is compared.

**Conclusions:**

A deformation gradient tensor estimator is developed based on a particle tracking technique and a least squares routine. Theoretical error bounds on the estimator are verified by numerical simulations, and the method’s capability is confirmed by physical experiments in evaluating large deformation and rotation near a free boundary. The proposed estimator is expected to open a door towards future material tests and experimental mechanics studies, especially in large deformation and large rotation scenarios.

## Introduction

Material deformation is the most essential kinematic process in solid mechanics, and its quantification remains a cornerstone of experimental mechanics. From the point-wise strain gauge [1] to state-of-the-art full-field measurement techniques [2], material deformation has been represented using linearized strain tensor components in a wide variety of cases. Traditionally, this has provided an accurate picture of small deformations in stiff engineering materials. However, the deformation gradient tensor, $\textbf{F}$, can give a more complete description of material deformation, particularly for a material undergoing large deformation, which occurs often in the case of elastomers [3] or hydrogels [4, 5]. $\textbf{F}$ can be used to separate material rotation and stretch via polar decomposition [6], thereby separating rigid-body rotation, which does not cost any strain energy, from the stretch, which does. Furthermore, when materials undergo local rotation, $\textbf{F}$ avoids mis-measurement of the linearized strain components that can be altered by the large rotation, and deviate from the material’s physical deformation states [7, 8].

**Fig. 1:**
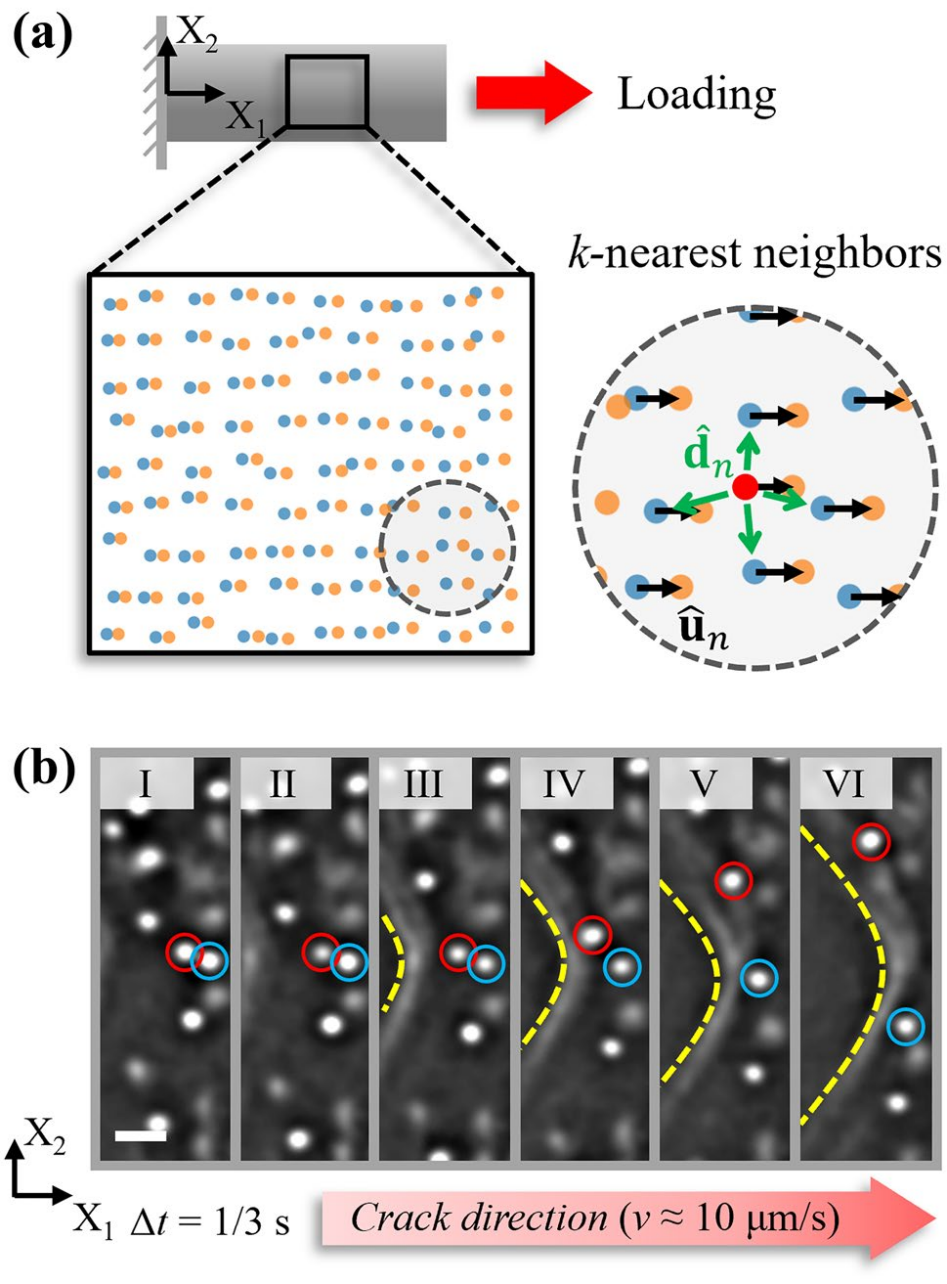
(**a**) A typical example of material deformation - schematic of a uniaxial tensile test of a bar with embedded particles. Particles are randomly distributed in the material in the reference state, indicated by blue dots. Upon loading, the material deforms and the particles are displaced accordingly, through a displacement vector ($\hat{\textbf{u}}_n$, black arrows) to their current locations (orange dots). For an arbitrary particle, e.g., the red particle shown in the inset, its *k* nearest neighbor particles can be found, with a relative position $\hat{\textbf{d}}_n$ indicated by the green arrows. (**b**) A time series of crack propagation during a physical experiment. With careful loading, the crack, indicated by yellow dashed line, propagates slowly at a velocity around 10 μm/s from left side to right side. Dispersed particles are effectively tracked. For example, the two particles, encircled in red and blue, are tracked very close to the crack tip despite their large displacements. The scale bar in frame I corresponds to 10 μm

In order to quantify $\textbf{F}$, as is shown by way of example for the uniaxially loaded bar in Fig. 1(a), the measurement of displacements at material points throughout the loading process is imperative [6]. Imaging methods have the distinct advantage of generating full-field displacement measurements. These methods include electronic speckle pattern interferometry (ESPI) [9], Moiré interferometry [10, 11], and digital image correlation (DIC) [2, 12, 13]. Among these methods, DIC stands out due to its facile experimental setup, robustness to environmental noise, and high flexibility vis-à-vis the selection of a measurement range [12–15].

In DIC, displacement data is obtained at gridded points by correlation of pixel subsets in speckle images before and after deformation. In order to obtain the displacement data, a warping vector consisting of stretches, shears and rigid translations is often used to optimize the correlation; however, oftentimes, rotation of a subset is not included in the warping vector. This may generate inaccuracies in the measured displacement data. Nevertheless, the grid format of displacement measurements enables elementary gradient computations with finite difference methods, rendering the calculation of $\textbf{F}$ simple. Despite its experimental and analytical convenience, implementation of DIC is challenging when a material undergoes large rotation or deformation, as can occur at a free interface, or near a crack tip. The challenge in implementing DIC under such circumstances arises due to the assumption of small changes in a subset’s orientation and shape [16]. In order to overcome this limitation, a wide range of strategies have been proposed, such as the ring template [17], polar coordination [18], a deformation transfer scheme [19], quasi-conformal mapping [20], scale-invariant feature transform (SIFT) [21–23], and speeded-up robust features (SURF) [24, 25]. Among these methods, the last two feature-based methods, are the most accepted in the implementation of DIC for initial guess improvement, due to their high robustness [25]. However, a sufficiently dense distribution of feature points in high quality images is required in these methods, and false matches cannot be completely eliminated [20]. Incremental DIC has also been used for the measurement of large deformation, whereby the reference images are updated throughout the process, thus reducing the decorrelation error [26, 27], but this inevitably leads to error accumulation Boundary discontinuities raise additional difficulties in the implementation of DIC, as occurs for cracks [28] or shear bands [29]. Although methods of discontinuity identification [30, 31], subset splitting [16, 32], and mesh-based methods [33–35] have been suggested, problems of low-error tolerance and heavy dependence on the accuracy of the initial guess or the crack recognition make error quantification challenging.

As an alternative to DIC, particle tracking measures displacement fields via dispersed particles. While this method is less popular, it inherently avoids the subset decorrelation and splitting problems in DIC, and enables reliable evaluation of large deformation and large rotation, especially near free surfaces. When correctly implemented, it can be highly accurate, achieving sub-pixel resolution of the particle positions [36–44]. Based on an image sequence of particles, the particle tracking algorithm can be generally implemented with two steps: particle locating, and particle linking [36]. By spatially locating particles in an individual frame and temporally linking across consecutive frames (e.g., particles tracked during crack propagation shown in Fig. 1(b)), particle trajectories are tracked and their displacements obtained. Depending on the imaging system, particle tracking can be extended from 2D to 3D [41–43, 45], realizing non-contact full-field measurements in both macro-scale and micro-/nano-scale in a manner similar to DIC [46–48].

Most developments and applications of particle tracking in the last three decades are found in biophysics [36, 42, 49–51], where particle tracking is used extensively for micro-rheology with the help of the mean squared displacement (MSD) of particles. The primary reason for this application is the simplicity of the initial guess for linking particles - as they undergo Brownian motion, their expected subsequent location in a given timestep is identical to their initial location. In the mechanics community, particle tracking has been applied to measure velocity in particle tracking velocimetry (PTV) for experimental fluid mechanics [41, 52–54]. When compared to the correlation-based particle image velocimetry (PIV) [55], PTV is more advantageous, as it yields increased spatial resolution and decreased computational cost [38, 39, 54]. The main factor that hinders further development and popularity of particle tracking is the non-gridded data format arising from the random distribution of particles. Randomly sampled data poses a challenge to gradient estimation. Even though local least-squares fitting of displacement fields and interpolation from irregular data to a regular grid have been reported [45, 56], performance of the methods under large gradients is unclear, and the related estimation errors have not been completely characterized.

In this manuscript, a full-field method for estimating the deformation gradient tensor is proposed, based on the non-gridded displacement data obtained from particle tracking in a manner similar to recent work [56]. At each particle location, the deformation gradient tensor is estimated from the displacement measurements of the given center particle and its *k*-nearest neighbors (see the inset of Fig. 1(a)). A detailed derivation of the deformation gradient estimator is given in “Estimator Derivation and Theory of Estimator Error” section, followed by an analysis of the estimator’s finite difference (FD) and measurement errors. In “Numerical Experiments and Parameter Sensitivity of the Finite-Difference and Measurement Error” section, the performance of the estimator is assessed via numerical simulations. Indeed, its error is computed as a function of parameters using numerically generated particle location data. The estimator is then employed in physical experiments in “Experimental Results” section, where its accuracy is validated with a uniaxial tensile test of a hydrogel sample. Furthermore, its effectiveness in large deformation/rotation measurement adjacent to a free boundary is confirmed by a mode I fracture experiment. These data are then directly compared with a DIC measurement of the displacement data using the same raw images. The relative merits of the particle tracking method and the proposed estimator are evaluated in the discussion of the results. With the advantages of full-field non-contact measurement, flexible selection of measurement range and resolution, inexpensive computational cost, and easy extension to 3D, the proposed particle tracking-based estimator and error quantification may open the door for future experimental mechanics study and material tests, broadening the tools available to the experimental mechanics community.

## Estimator Derivation and Theory of Estimator Error

### Estimator Derivation

In order to compute the deformation gradient $\textbf{F}(\textbf{r})$ at a particle’s location $\textbf{r}_0$ in the reference state, the particle’s displacement upon applied deformation, $\textbf{u}_0$, and those of its *k*-nearest neighbors (located at $\textbf{r}_1,\dots ,\textbf{r}_k$) are first evaluated, as shown in Fig. 1(a). $\textbf{F}(\textbf{r}) = \mathbf {\nabla u(r) + I}$ quantifies the variation of the displacement field across infinitesimal distances, where the displacement of the $n^\text {th}$ particle is given by $\hat{\textbf{u}}_n$, for *n* ranging from 0 to *k*.

Assuming that the *k*-nearest neighbors, particles $1,\dots ,k$ are close to particle 0, a first-order Taylor expansion of displacement closely approximates the $n^\text {th}$ particle’s displacement:

1 $$\begin{aligned} \textbf{u}(\textbf{r}_n) \approx \textbf{u}(\textbf{r}_0) + \mathbf {\nabla u}(\textbf{r}_0)^\textrm{T} (\textbf{r}_n - \textbf{r}_0) \end{aligned}$$

The values of the displacement field at particle locations are replaced with measurements from experiments, and a least squares estimate of $\mathbf {\nabla u}(\textbf{r}_0)$ is computed over all *k* equations. Summing with the identity tensor produces an estimate of $\textbf{F}(\textbf{r}_0)$ as follows:

2 $$\begin{aligned} \hat{\textbf{F}}(\textbf{r}_0)&= {{\underset{\textbf{G}}{\text {argmin}}}} (\sum _{n=1}^k \Vert \hat{\textbf{u}}_n - \hat{\textbf{u}}_0 - \textbf{G}^\textrm{T} \textbf{d}_n \Vert ^2) \\ &\quad+ \textbf{I}= (\sum _{n=1}^k \textbf{d}_n \textbf{d}_n^\textrm{T})^{-1} \sum _{m=1}^k \textbf{d}_m (\hat{\textbf{u}}_m^\textrm{T} - \hat{\textbf{u}}_0^\textrm{T}) + \textbf{I}, \end{aligned}$$

where $\textbf{G}$ is the argument which minimizes the term $\sum _{n=1}^k \Vert \hat{\textbf{u}}_n - \hat{\textbf{u}}_0 - \textbf{G}^\textrm{T} \textbf{d}_n \Vert ^2$, $\textbf{d}_n = \textbf{r}_n - \textbf{r}_0$ is the relative position of the $n^\text {th}$ neighbor, and $\Vert .\Vert$ denotes the Euclidean norm.

Note that none of the dyadic tensors $\textbf{d}_n \textbf{d}_n^\textrm{T}$ are invertible. Indeed, when $k=1$, displacement variation is measured only along a single direction; thus, the gradient cannot be inferred along the perpendicular plane. As a result, a minimum number of neighbors, equal to the number of spatial dimensions, is required to estimate the gradient. The stochastic nature of particle positions realized in experiments ensures that the sum of tensors $\sum _{n=1}^k \textbf{d}_n \textbf{d}_n^\textrm{T}$ is almost surely invertible - indeed, in three dimensions with 3 or more particles, the particles would have to be aligned perfectly for this not to be the case. This occurs with probability zero.

To estimate $\textbf{F}$, a reference set of $\textbf{d}_n$ must be measured before deformation occurs, representing the undeformed material. In time-dependent experiments, these $\textbf{d}_n$ are reused for measurement of the deformation gradient at each time step. Noise in the initial measurement of $\textbf{d}_n$ thus affects all estimates. Inaccuracies arising from noise in the reference set of $\textbf{d}_n$ can be minimized by oversampling - recording a series of frames prior to deformation which can be used to reduce the noise in the measured value of $\textbf{d}_n$. The anticipated noise due to a single measurement of $\textbf{d}_n$ would be limited by the resolution of the particle’s location in the particle tracking algorithm. Typically, the point-spread function of particles can be used to achieve subpixel resolution of particle locations; for an average inter-particle spacing of $\sim 40$ pixels, this corresponds to an error of approximately 1% in the magnitude and direction of $\textbf{d}_n$.

### Theory of Estimator Error

In “Estimator Derivation” section, one important assumption underpinned the estimator of equation (2). Inter-particle distances were assumed to be small for the *k*-nearest neighbors. This is consistent with neglecting the higher order terms of the Taylor expansion of displacement, and in practice, produces an approximation error referred to as ‘finite difference error,’ $\varepsilon _\text {FD}$. Error in the measurements of particle positions constitute a second source of error, denoted by $\varepsilon _\text {M}$.

In Appendix 1, it is shown that assuming independent Gaussian measurements $\hat{\textbf{u}}_n \sim \mathcal {N}(\mathbf {u(r}_n),\frac{\sigma ^2}{3}\textbf{I})$ and that one does not use nearest neighbors across cracks or sharp lobes, the estimator’s mean square error (MSE) can be expanded as:

3 $$\begin{aligned} \mathbb {E}[\Vert \hat{\textbf{F}(r}_0) - \mathbf {F(r}_0) \Vert _F^2 \mid \{\textbf{d}_n\}_{n=1}^k ] = \varepsilon _\text {FD}^2 + \varepsilon _\text {M}^2, \end{aligned}$$

where $\varepsilon _\text {FD}$ and $\varepsilon _\text {M}$ are given by

4a $$\begin{aligned}&{\varepsilon _\text {FD} = \frac{R}{2} \sqrt{\sum _{i=1}^3 \Vert \frac{1}{k} \sum _{n=1}^k \frac{\textbf{d}_n^\textrm{T}}{R} \nabla ^2 u_i(\varvec{\xi }^i_n) \frac{\textbf{d}_n}{R} \, \mathbf {A^{-1}} \frac{\textbf{d}_n}{R} \Vert ^2}} , \end{aligned}$$

4b $$\begin{aligned}&{\varepsilon _\text {M} = \frac{\sigma }{R \sqrt{k}}\sqrt{\text {tr}(\textbf{A}^{-1}) + k \Vert \mathbf {A^{-1}} \frac{1}{k}\sum _{n=1}^k \frac{\textbf{d}_n}{R} \Vert ^2}}, \end{aligned}$$

where $\textbf{A} = \frac{1}{R^2 k} \sum _{n=1}^k \textbf{d}_n \textbf{d}_n^\textrm{T}$, $\varvec{\xi }^i_n$ is the location along the line connecting the $0^\text {th}$ and $n^\text {th}$ particle at which the second-order Taylor expansion generates the exact displacement component $u_i$. $\Vert .\Vert _F$ denotes the Frobenius norm.

Both errors $\varepsilon _\text {FD}$ and $\varepsilon _\text {M}$ depend on $\{\textbf{d}_n\}_{n=1}^k$. While the $\textbf{d}_n$ are random, they are measured quantities, and can be used to compute $\varepsilon _\text {M}$ from experimental data. On the other hand, computing $\varepsilon _\text {FD}$ requires knowledge of second-order derivatives of displacement, which are unknown in practice.

Under the hypothesis of independent, unbiased Gaussian measurements, the estimator given in equation (2) is the minimum mean square error estimator (MMSE) in the sense that it minimizes $\varepsilon _\text {M}$.

## Numerical Experiments and Parameter Sensitivity of the Finite-Difference and Measurement Error

**Fig. 2:**
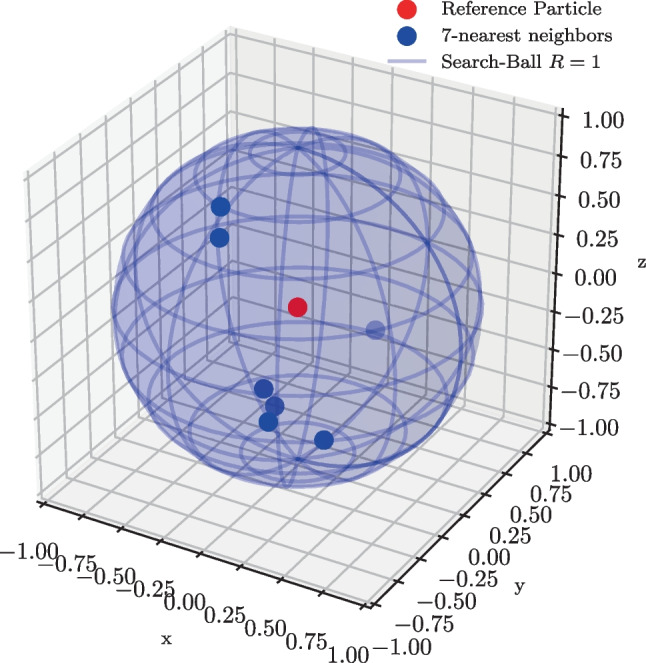
Instance of numerically simulated data: the position of 7 neighboring particles is sampled uniformly at random inside sphere of $R=1$. The position of each particle is made inaccurate by applying Gaussian noise

A series of numerical simulations are run to investigate the performance of the estimator given in equation (2). In each instance, *k* neighbors are sampled uniformly at random inside a sphere of radius *R* around a center particle at $\textbf{r}_0$, as shown schematically in Fig. 2. A displacement field is prescribed to all particles. Noise sampled from $\mathcal {N}(\textbf{0},\frac{\sigma ^2}{3}\textbf{I})$ is added to the displacement field values at each particle location. The displacement measurements resulting from the data generated in this fashion are used to estimate the deformation gradient at $\textbf{r}_0$.

As mentioned in “Estimator Derivation and Theory of Estimator Error” section, error arises from both the FD approximation and the measurement. In order to evaluate these two sources of error systematically, two sets of simulations are run.

**Fig. 3:**
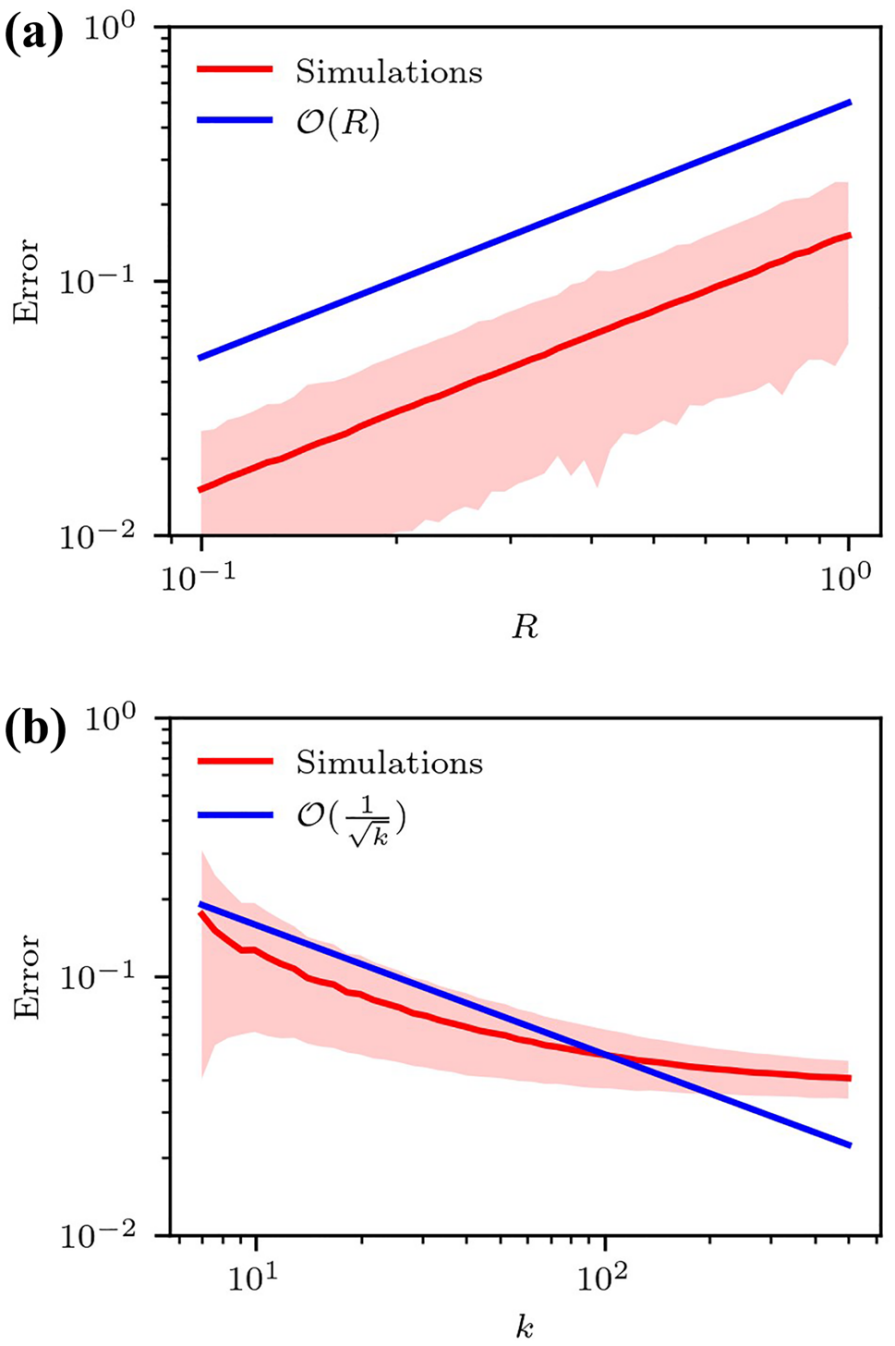
Relative finite difference error as computed in equations (4a) and (5) of the $\textbf{F}$-estimator for a sinusoidal displacement field $\mathbf {u(r)} = (\text {cos}(\varvec{\omega }\cdot \textbf{r} + \phi ), \text {sin}(\varvec{\omega }\cdot \textbf{r}+\phi ),1)$, and zero measurement noise, averaged over 10,000 instances. The shaded area indicates one estimated standard deviation’s width from the mean for (**a**) $k = 7$, $\sigma = 0$ and (**b**) $R = 1$, $\sigma = 0$. Blue lines indicate the scaling identified in the legend as a guide to the eye

**Fig. 4:**
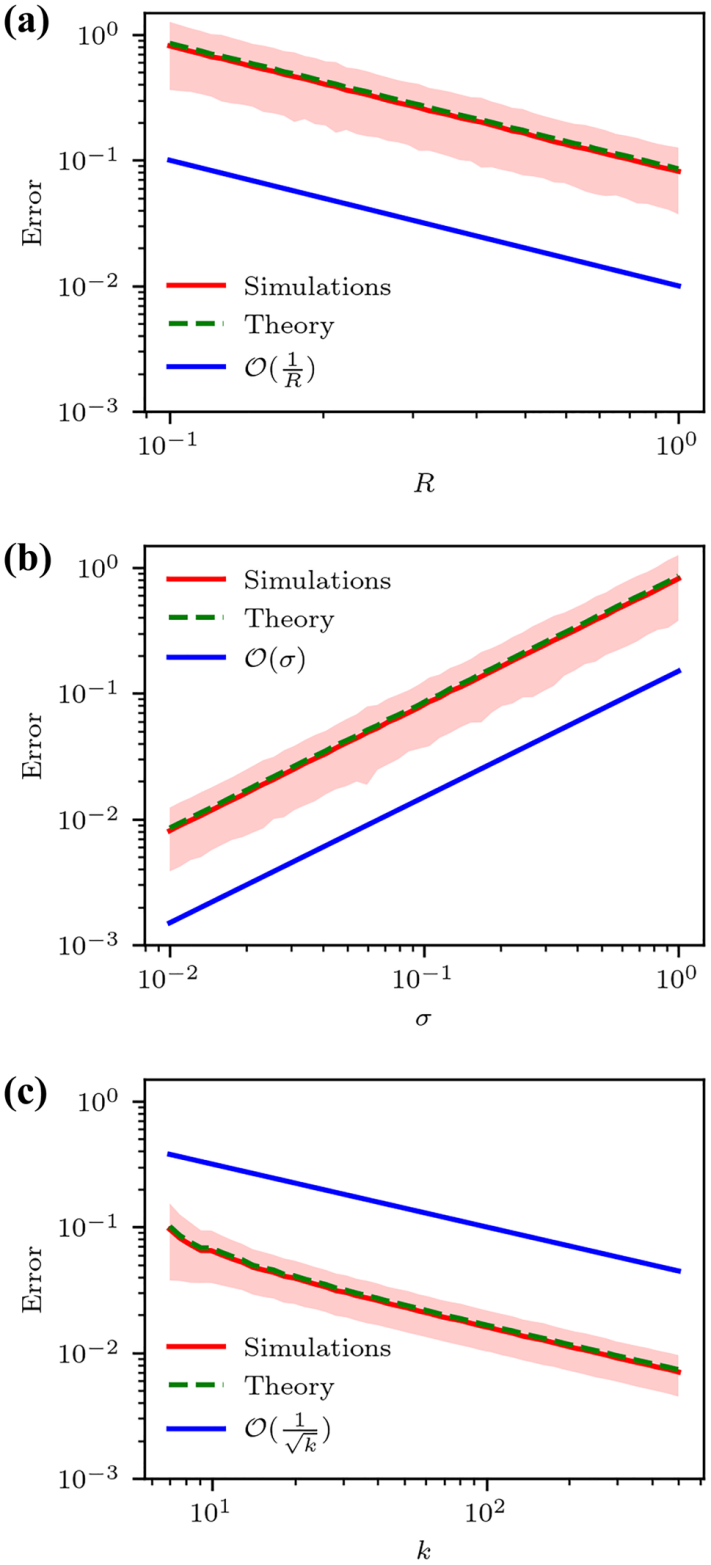
Relative measurement error as computed in Eqs. (4b) and (5) of the $\textbf{F}$-estimator for a linear displacement field $\mathbf {u(r) = r}$, averaged over 10,000 instances. The shaded area of one estimated standard deviation’s width from mean (**a**) $\sigma = 0.1$, $k=7$, (**b**) $R = 1$, $k = 7$, and (**c**) $R = 1$, $\sigma = 0.1$. Blue lines indicate the scaling identified in the legend as a guide to the eye

In both batches of simulated data, error is averaged over 10,000 instances for each triplet of values $(k,R,\sigma )$. This generates the red curves in Figs. 3 and 4. Relative error is defined as:

5 $$\begin{aligned} \Delta = \frac{\Vert \mathbf {F(r}_0) - \hat{\textbf{F}(r}_0)\Vert _F}{\Vert \mathbf {F(r}_0)\Vert _F} \end{aligned}$$

In physical experiments, *k* and *R* are related by the particle density; however, using the simulated data, we vary them independently to evaluate the estimator’s sensitivity to these parameters.

The first batch of numerical experiments is simulated without measurement noise in order to isolate FD error. A sinusoidal displacement field is prescribed with frequency $\Vert \varvec{\omega } \Vert = 1$, and uniformly random direction and offset,

6 $$\begin{aligned} \mathbf {u(r)} = \begin{pmatrix} \text {cos}(\varvec{\omega } \cdot \textbf{r} + \phi ) \\ \text {sin}(\varvec{\omega } \cdot \textbf{r} + \phi ) \\ 1 \end{pmatrix}. \end{aligned}$$

The sinusoidal displacement field is selected to ensure that the second-order derivatives, and thus the error, are bounded.

As seen in Fig. 3(a), for fixed *k*, $\mathcal {O}(R)$ dependence is observed. *R* does not appear to affect the standard deviation. Meanwhile, for fixed *R*, error decreases with respect to *k* as shown in Fig. 3(b) before seemingly reaching a nonzero asymptotic limit.

In the next set of numerical experiments, measurement noise is introduced. Instead of a sinusoidal field, linear displacements are prescribed on the particles so as to negate FD error,

7 $$\begin{aligned} \mathbf {u(r) = r}. \end{aligned}$$

In addition to relative error of the deformation gradient, the expected error $\varepsilon _\text {M}$ is also reported. It is computed at each instance, and then averaged.

In Fig. 4(a) and (b), error rates obey $\mathcal {O}(\frac{1}{R})$, and $\mathcal {O}(\sigma )$. Neither *R* nor $\sigma$ affect the standard deviation of the error. Furthermore, for fixed *R* and $\sigma$, error is seen to decrease as $\mathcal {O}(\frac{1}{\sqrt{k}})$ in Fig. 4(c). The expected measurement error $\varepsilon _\text {M}$ is very close to the error observed in simulations after averaging over simulation instances.

Numerical simulations confirm the first-order nature of the FD error of the estimator given by equation (2). Moreover, the behavior of $\varepsilon _\text {M}$ reveals $\frac{1}{\sqrt{k}}$ filtering of measurement noise; this is typically observed in unweighted averaging. In addition, signal-to-noise ratio (SNR) is expressed in the $\frac{\sigma }{R}$ dependence. When neighbors are within $\approx \sigma$ distance of the center particle, measurement noise can completely overwhelm measurement of the direction. As a result, it may be beneficial to omit very close neighbors in gradient computations. Larger *R* leads to more robust measurements, but also penalizes the linear approximation through an increased FD error. This constitutes an intrinsic trade-off between measurement and FD errors. The optimal choice of *R* or *k* depends on the displacement fields and the magnitude of measurement noise. For large curvatures on the scale of *R*, the bulk of the error arises from FD approximation, suggesting the optimal value of *R* might be smaller, provided *k* remains sufficiently large, e.g. $\ge 7$. In physical experiments, *k* and *R* are related through the numerical density of the particles $\rho$. The theoretical computations of measurement error match those of simulations, underscoring the accuracy of the theoretical expressions.

## Experimental Results

**Fig. 5:**
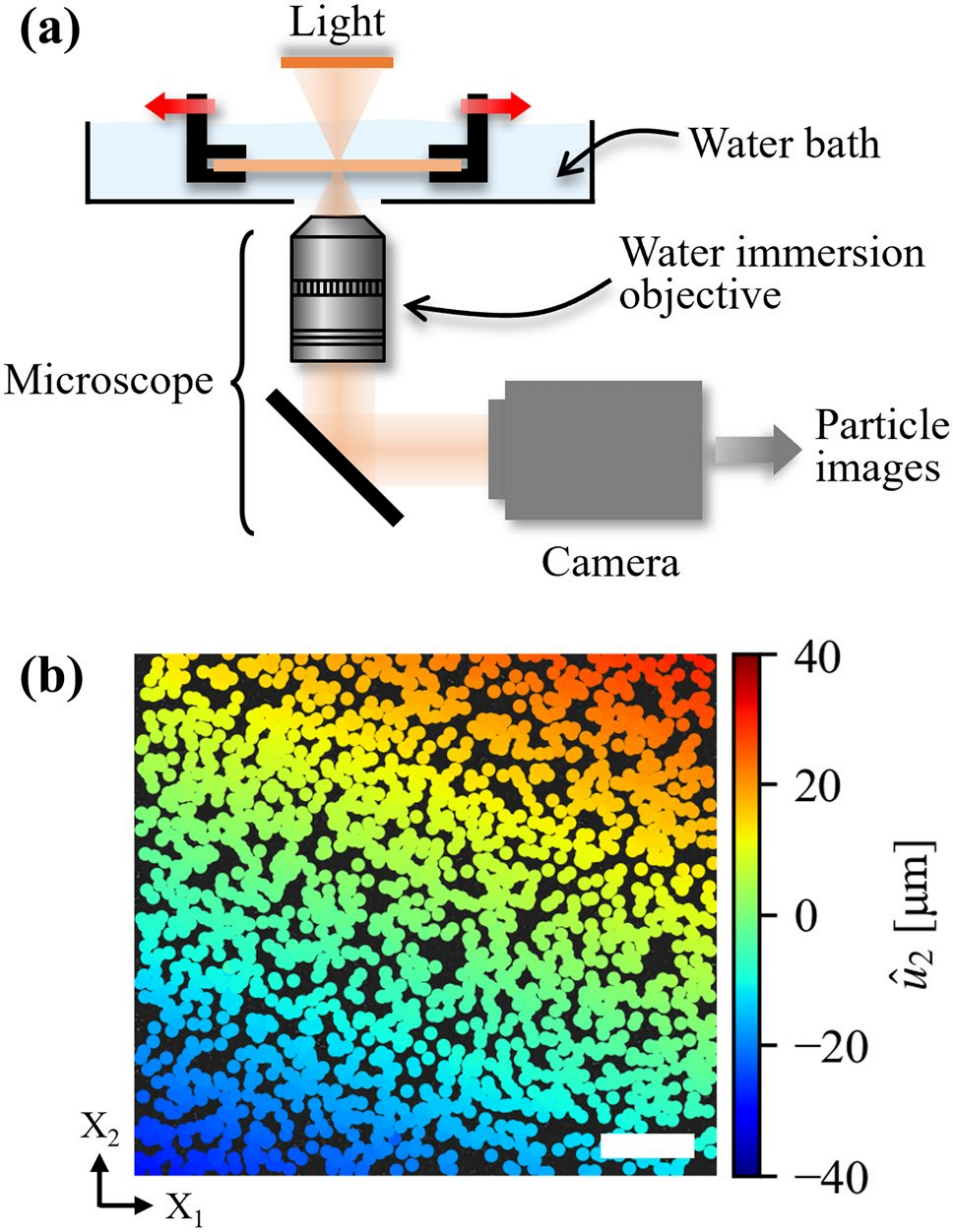
(**a**) Experimental setup. The hydrogel sample is clamped by two grips which are symmetrically actuated by a servo motor used to apply uniaxial tensile loading. The sample is illuminated using a diascopic dark-field light source. Throughout the experiment, the sample remains immersed in a water bath. The light scattered by embedded particles is imaged onto the sensor of a high-resolution camera through a microscope via a $10\times$ water-immersion objective. (**b**) The displacement field along loading direction, $\hat{u}_2$, under uniaxial tensile loading, is depicted. Particles are tracked densely throughout the field-of-view. The smoothness of the displacement distribution assures that tracking is accurate. The scale bar is 100 µm

To evaluate the accuracy and effectiveness of the proposed deformation gradient estimator, three physical experiments - 2D and 3D uniaxial tensile tests, and a mode I fracture test - are carried out on the micro-scale testing apparatus shown in Fig. 5(a). The samples used in these experiments are polyacrylamide hydrogels, which are prepared based on the recipe that has been widely used for fracture experiments [5, 57, 58]. Micro polystyrene particles (diameter $1.1 \pm 0.1$ µm, Sigma-Aldrich) are mixed with the hydrogel solution at a concentration of $0.005\ \mathrm {wt.} \%$. The hydrogel is polymerized between two glass plates separated by 190 µm spacers, and immersed in water for more than 24 h to reach equilibrium before cutting into 1 cm wide samples. The samples are immersed in a water bath and illuminated with a dark-field scheme. The samples are tightly clamped by two grips which are symmetrically actuated by a servo motor for exerting tensile loading. A microscope (Nikon eclipse Ti, tube lens magnification: 1.5$\times$), equipped with a $10\times$ water-immersion objective (Nikon Plan Fluor 10$\times$/0.30W) and a high-resolution camera (Hamamatsu C13440, resolution: 2048 × 2048 pixels, bit depth: 16 bit), is used to record the images (image stacks for 3D) of the particles embedded in the transparent hydrogel. The use of the water-immersion objective ensures high fidelity imaging of the particles by minimizing the refractive index mismatch between the hydrogel and objective.

The procedure of estimating the deformation gradient tensor from particle images is shown in Algorithm (1). The particle images are first pre-processed by a bandpass filter to suppress image noise and to remove any large structures, which might be falsely detected as a particle during the tracking process. The filtered images are then used as inputs for the open-source particle tracking algorithm, Trackpy [59], which accurately locates particles in individual frames, and then correctly links them to their trajectories in consecutive frames. Discrete displacement fields can be inferred from particle trajectories, and the deformation gradient tensor at each particle location can be computed by the proposed estimator given in equation (2). Continuous fields can be obtained from the particle tracking data by interpolation over the desired field at each particle. Furthermore, other deformation tensors, e.g., finite strain tensor, can be conveniently computed according to their definitions from continuum mechanics theory [6].

**Table:** 

**Algorithm 1** Deformation gradient tensor estimation based on particle tracking
**Input:** Image sequence
**Output:** Deformation gradient tensor and derivative tensors, i.e., rotation tensor, stretch tensor, strain tensors.

** Phase 1 - Particle tracking**
+ Image pre-processing (optional)
+ Find particle locations in individual images
- Input an estimate of particle diameter
- Filter out spurious particles
- Check sub-pixel accuracy
+ Link particles in consecutive images

** Phase 2 - Estimation of deformation gradient tensor**
+ Construct displacement fields from particle trajectories
+ Loop over all particles
- Find *k* nearest neighbor particles and their relative positions
- Perform finite difference with estimator (2)

** Phase 3 - Calculation of other deformation tensors (optional)**
+ Polar decomposition $\rightarrow$ stretch tensor and rotation tensor
+ Deformation tensor $\rightarrow$ finite strain tensor
+ Constitutive model $\rightarrow$ stress tensor

### 2D Uniaxial Tensile Test

A uniaxial tensile test is conducted first to verify accuracy of the proposed estimator since the displacement field and deformation gradient tensor are well known for linear elastic solids under the assumption of small strains as:

8 $$\begin{aligned} \mathbf {u(r) = D r + c}, \end{aligned}$$

and

9 $$\begin{aligned} \mathbf {F(r)=\nabla u(r) + I = D + I}, \end{aligned}$$

where $\textbf{D}$ is a constant, $2\times 2$ matrix representing the constant deformation and $\textbf{c}$ is $2\times 1$ vector representing the rigid displacement. For such a linear displacement field, the FD error is zero, as it is fully described by the first order Taylor expansion in equation (1).

While loading the sample, particle images are recorded at each strain increment. By tracking the particles before and after deformation, the displacement fields are measured, e.g., the displacement component along the loading direction, $\hat{u}_2$, as shown in Fig. 5(b). The displacement fields yield the displacement gradient tensor $\hat{\textbf{D}}_\text {fit}$ by fitting a plane to equation (8) using a least squares routine, and thus the deformation gradient tensor, $\hat{\textbf{F}}_\text {fit}$. Once $\hat{\textbf{F}}_\text {fit}$ is determined, on the one hand, the uniaxial strain, $\varepsilon _{22}$, is easily calculated with continuum mechanics theory as $6.14\%$, confirming the small strain assumption; on the other hand, the small mean squared errors $\text {MSE}(\hat{u}_{1}-u_{1, \text {fit}})=0.15$ pixel and $\text {MSE}(\hat{u}_{2} -u_{2, \text {fit}})=0.37$ pixel from plane fitting of $\hat{u}_1$ and $\hat{u}_2$ demonstrate the high accuracy of the fitting, and enable the FD error-free $\hat{\textbf{F}}_\text {fit}$ to be used as a reference for evaluation of our deformation gradient estimator.

**Fig. 6:**
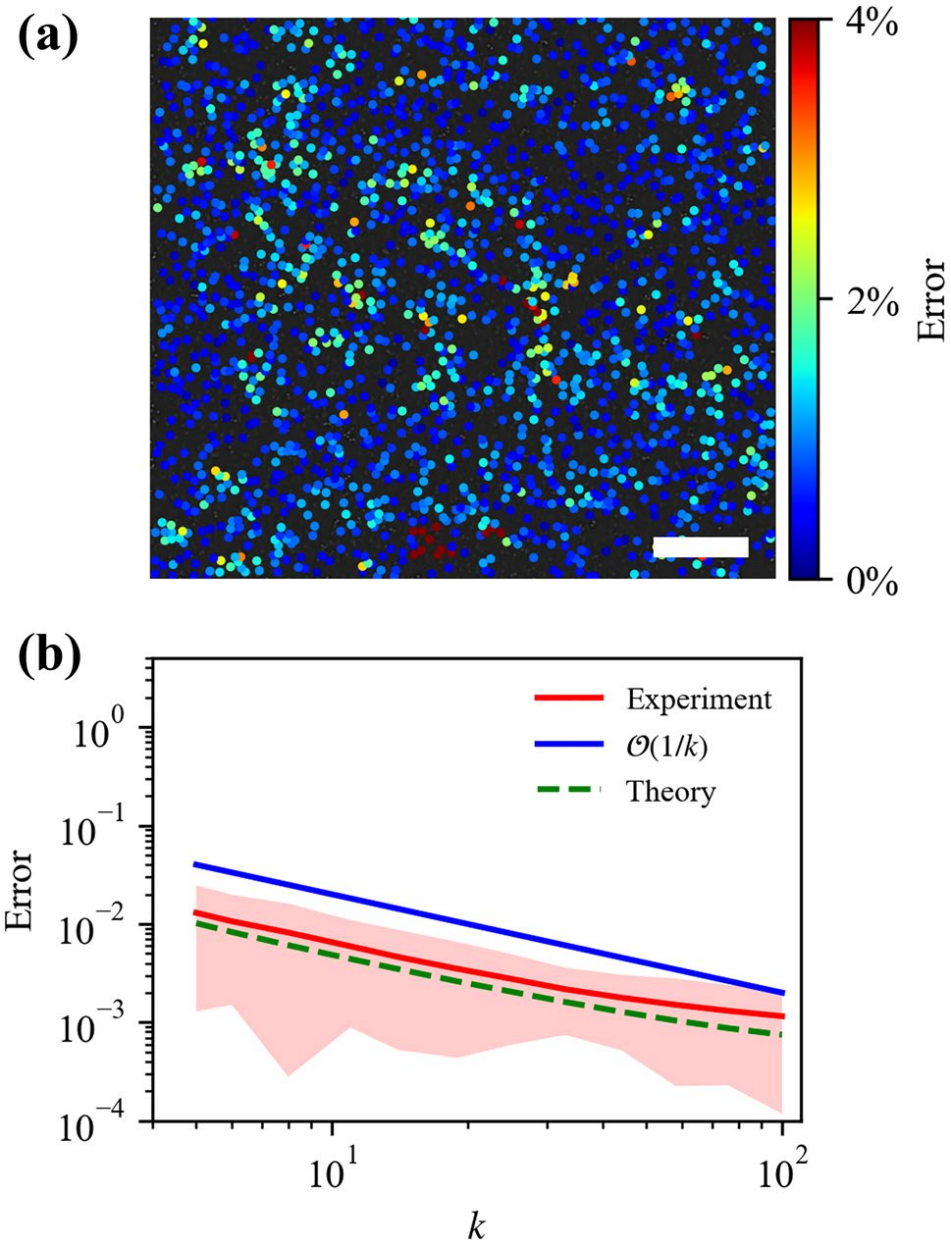
(**a**) Relative Frobenius error between the estimated $\hat{\textbf{F}}$ and the spatially averaged $\textbf{F}_{th}$ obtained by fitting a plane to the displacement fields. The large area with near-zero relative error highlights the accuracy of the proposed estimator. (**b**) Experimental and theoretical relative error is calculated as a function of the number of neighboring particles used for the estimation, *k*. The red solid line is the average experimental relative error for all particles, and the red shaded area indicates one standard deviation. The green dashed line is plotted according to equation (4b), and the blue solid line $\mathcal {O}(1/k)$ is plotted as a guide to the eye

The deformation gradient tensor, $\hat{\textbf{F}}$, is computed using the estimator given by equation (2) at each discrete particle by selecting a given number of nearest-neighbor particles, *k*. The relative error between $\hat{\textbf{F}}$ and $\hat{\textbf{F}}_\text {fit}$ is calculated according to equation (5), and is shown in Fig. 6(a) where $k=7$ is used for estimation. For most particles, the relative error remains near-zero and indicates the high accuracy of our proposed estimator; some particles show a larger discrepancy (maximum difference of $4\%$); this might arise from the non-uniform distribution of particles, or local incorrect linking. The relative error is evaluated for a varied *k*, and compared to the theoretical measurement error in equation (4), as shown in Fig. 6(b). The relative error is observed to be larger than the theoretical prediction throughout the range of *k* from 5 to 100, which may arise due to the least squares fit of displacement, or due to inaccuracies in the measurement of the reference state. Furthermore, the relative error is found to decrease at a rate of $\mathcal {O}(\frac{1}{k})$ until saturation at very large values of *k* due to the finite domain size. The $\mathcal {O}(\frac{1}{k})$ rate can be understood by considering the error rate of $\mathcal {O}(\frac{\sigma }{R \sqrt{k}})$ derived from numerical simulations and the fact that *R* is related to *k* via the 2D particle density $\rho$, $R = \sqrt{k/\rho \pi }$, which yields an error dependence $\mathcal {O}(\frac{1}{k})$ as observed experimentally. The reduced rate of decrease in the large-*k* limit results from the finite size of the domain. Nevertheless, a degree of consistency is established between theory and experiment, and effectiveness of the estimator proposed in equation (2) is confirmed.

### Mode I Fracture Test

Our proposed estimator was also used to measure the deformation gradient near a mode I crack tip, where large deformation and rotation occur. The experiment is carried out with the same experimental setup depicted in Fig. 5(a) and a similar particle-embedded hydrogel sample. A notch is pre-cut at the center of one edge of the sample, and it propagates slowly upon careful application of remote tensile loading. Images of particles are recorded before the crack is hundreds of micrometers away from field-of-view at 3 frames per second. The recorded particle images are bandpass-filtered, and tracked with Trackpy [59]. The particles are first located in individual frames with an estimated diameter of 19 pixels, and then linked to their trajectories. Considering the substantial displacements near the crack tip between consecutive images, a velocity-based prediction function is employed for robust particle linking. An adaptive search regime is also applied so as to balance the number of candidate particles, and avoid incorrect delinking (search range too small) or an overwhelming number of particle candidates (search range too large). As can be seen from the time series shown in Fig. 1(b), the particles are correctly tracked, even very close to the crack tip.

**Fig. 7:**
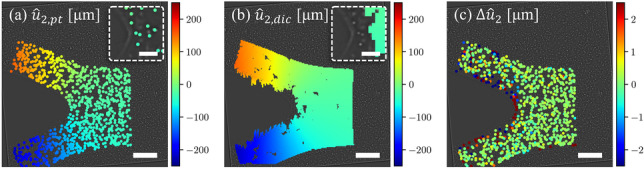
Displacement fields are measured in a Mode I fracture experiment of a particle-embedded hydrogel sample. Displacement fields along the loading direction, $u_2$, were measured by both particle tracking and DIC during crack propagation. The scale bars are 100 µm in each panel and 20 µm in the insets. (**a**) Displacement fields $\hat{u}_{2,pt}$ tracked at discrete particles. The particles are tracked robustly, and the obtained displacement is symmetric about the crack path. Inset: particles are tracked within 10 µm of the crack tip. (**b**) Displacement fields $\hat{u}_{2,dic}$ calculated with DIC. A grid with 10-pixel spacing is specified in the $1400 \times 800$ pixel region-of-interest (ROI) for DIC calculation using a circular subset with a 39-pixel radius. The displacement field $\hat{u}_{2,dic}$ appears as similar to the displacement field $\hat{u}_{2,pt}$ obtained by particle tracking; however, artifacts and gaps are readily identifiable near the crack surface as well as in the immediate vicinity of the crack tip. Correlation is not achieved at the crack tip, as shown in the inset. (**c**) The difference between $\hat{u}_{2,dic}$ interpolated at particle locations and $\hat{u}_{2,pt}$ determined by particle tracking

Therefore, the displacement fields are accurately retrieved, and the displacement component along the loading direction, $\hat{u}_{2,pt}$, is symmetric to the crack path, as shown in Fig. 7(a). The measured displacement is valid in the immediate vicinity of the crack tip, as can be seen in the inset of Fig. 7(a). For comparison, the open-source DIC software Ncorr [60] is used to calculate the displacement field with a 39-pixel subset radius and 10-pixel grid spacing. The obtained displacement data is filtered by a cutoff threshold of correlation coefficient (here we use 0.5) to ensure a reliable correlation. Although the particle images are not optimal for DIC analysis, the calculation parameters are optimized by numerous trial calculations, and the subset size is verified by the average sum of square of subset intensity gradients (SSSIG) [61] to ensure the accuracy of our DIC measurement. The displacement component, $\hat{u}_{2,dic}$, calculated with DIC is shown in Fig. 7(b), and its deviation from the particle tracking measurement, $\Delta \hat{u}_{2} = \hat{u}_{2,dic} - \hat{u}_{2,pt}$, is computed by interpolation of $\hat{u}_{2,dic}$ at particle locations; $\Delta \hat{u}_{2}$ is shown in Fig. 7(c). Away from the crack, the DIC and particle tracking measurements offer a very similar value of displacement, and the difference is near zero. While the agreement confirms the high accuracy of both methods, some spikes and voids are seen near the crack surface and crack tip in the DIC field, leading to a large discrepancy in these regions, as shown in Fig. 7(c). $\hat{u}_{2,dic}$ seems correct as it approaches the crack surface, but in fact, the correlation is unrealistic, because the subset is displaced outside the material boundary. Due to large deformation, subsets near the crack tip are characterized by unreliable correlation coefficients and filtered out, leaving voids in the data, as shown in the inset of Fig. 7(b). Accurate displacement measurement is fundamental for deformation gradient estimation, no matter which estimation approach is employed. Therefore, in the presence of free surfaces and large deformation or rotation, the particle tracking-based method is more reliable.

**Fig. 8:**
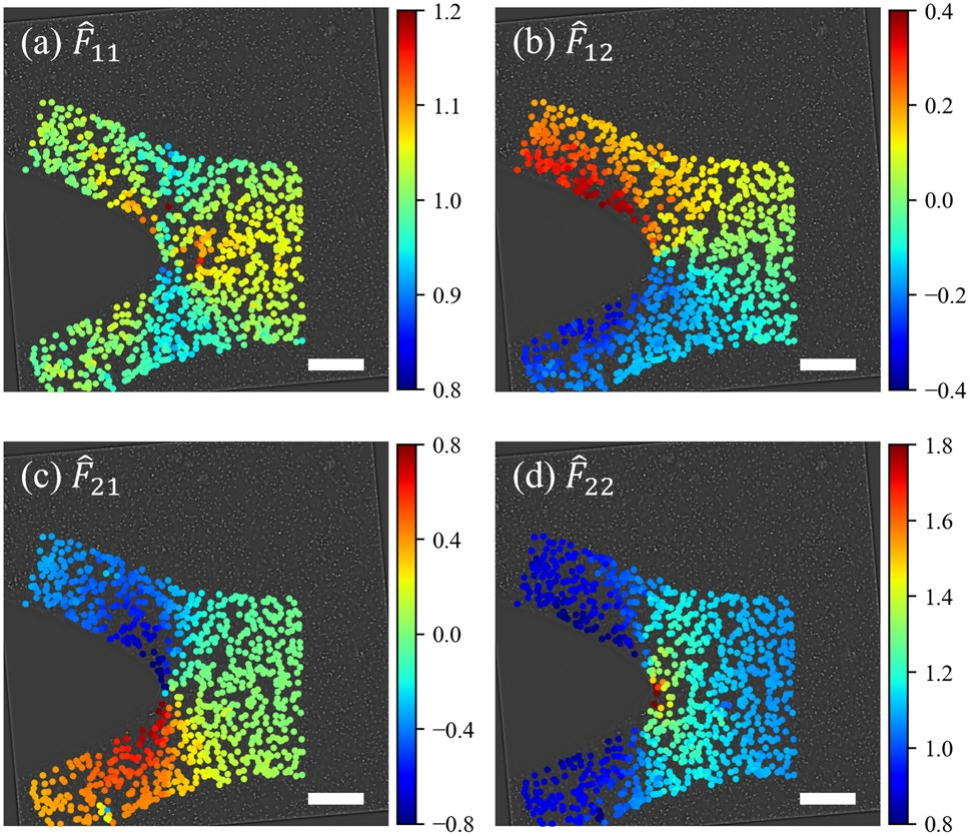
Components of deformation gradient tensor near a Mode I crack tip evaluated with the proposed estimator: (**a**) $\hat{F}_{11}$, (**b**) $\hat{F}_{12}$, (**c**) $\hat{F}_{21}$, and (**d**) $\hat{F}_{22}$. Note that the ranges of color bars are not identical for clear representation of the fields. The scale bar corresponds to 100 µm

Using our deformation gradient estimator given by equation (2), the deformation gradient tensor $\textbf{F}$ is estimated at each particle’s location with its 7 nearest-neighbor particles, as shown in Fig. 8. Components $\hat{F}_{11}$ and $\hat{F}_{22}$ are symmetric about the crack path while components $\hat{F}_{12}$ and $\hat{F}_{21}$ are anti-symmetric. The $\hat{\textbf{F}}$ estimated from the measured displacements resembles the identity tensor ahead of the crack tip in the far field. Approaching the crack tip, $\hat{\textbf{F}}$ strongly deviates from the identity tensor, as deformation becomes large; the dominant component is $\hat{F}_{22}$. In the post-crack regions, off-diagonal components, $\hat{F}_{12}$ and $\hat{F}_{21}$, are larger, especially near the crack surface. It is also visible in these regions that $\hat{F}_{22}$ is less than 1, as the deformation is evaluated relative to the first frame of the recorded images, where the sample is stretched compared to the relaxed state, which it comes close to attaining in the wake of the crack. To enable a rigorous study, an image of the particles in the material’s reference state should be included as the reference for particle positions, but maintaining focus on a same group of particles is challenging. Alternatively, the particle locations in the reference state could be estimated by calculating the uniaxial tensile stretch far ahead of the crack, where the stretch is approximately uniform [62]. Here, displacement is measured relative to the uniformly stretched configuration in the first frame for consistency when comparing to the strains measured from the DIC data.

**Fig. 9:**
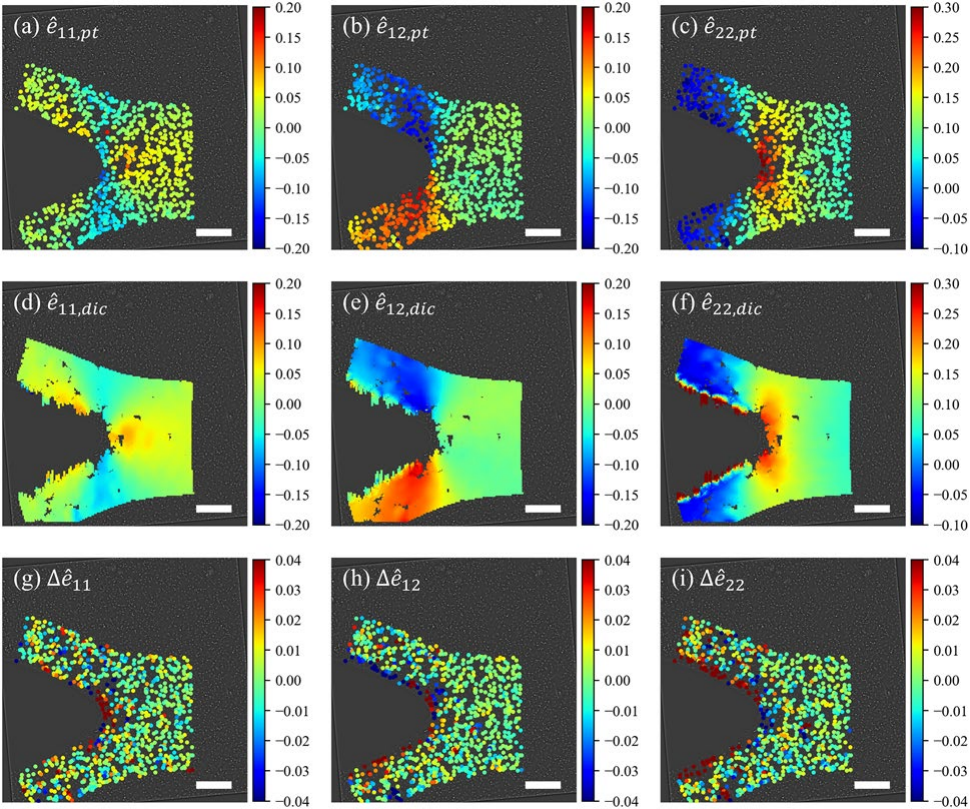
Comparison of Eulerian-Almansi finite strain tensors obtained from the particle tracking-based estimation and the DIC calculation. (**a**)–(**c**) Components of finite strain tensor $\hat{\textbf{e}}_{pt}$ obtained by particle tracking and the deformation gradient estimation: $\hat{e}_{11,pt}$, $\hat{e}_{12,pt}$, and $\hat{e}_{22,pt}$, respectively. (**d**)–(**f**) Components of finite strain tensor $\hat{\textbf{e}}_{dic}$ calculated from DIC: $\hat{e}_{11,dic}$, $\hat{e}_{12,dic}$, and $\hat{e}_{22,dic}$, respectively. (**g**)–(**i**) Difference of strain components measured by DIC and particle tracking: $\Delta \hat{e}_{11}$, $\Delta \hat{e}_{12}$, and $\Delta \hat{e}_{22}$, respectively, where $\Delta \hat{\textbf{e}} = \hat{\textbf{e}}_{dic} - \hat{\textbf{e}}_{pt}$. The scale bar corresponds to 100 µm

The Eulerian-Almansi finite strain tensor, $\hat{\textbf{e}}_{pt}$, is computed at each particle location using the estimated $\hat{\textbf{F}}$ via $\hat{\textbf{e}}_{pt} = (\textbf{I}-\hat{\textbf{B}}^{-1})/2$, where $\hat{\textbf{B}}$ is the left Cauchy-Green deformation tensor, $\hat{\textbf{B}} = \hat{\textbf{F}}\hat{\textbf{F}}^\textrm{T}$; these strain components are shown in Fig. 9(a)–(c). The same strain tensor, $\hat{\textbf{e}}_{dic}$, is evaluated using the DIC data by fitting a plane to the displacement fields in a local $5\times 5$-nodes window, as shown in Fig. 9(d)–(f). The difference between DIC measurement and our particle tracking-based estimation, $\Delta \hat{\textbf{e}} = \hat{\textbf{e}}_{dic} - \hat{\textbf{e}}_{pt}$, is further computed, and plotted in Fig. 9(g)–(i). It is observed that both methods measure similar strain distributions, with dominant tensile strain near the crack tip and substantial shear strain in the wake of the crack. The measured strain values are similar for all components in most of the ROI, as the near-zero difference suggests. An apparent discrepancy occurs near the crack surface and crack tip, resulting from a poor correlation when performing DIC in these regions. Near the crack surface, the strain measured from particle tracking-based estimation is smooth, but is jagged in the DIC measurement, even though the displacement fields are smooth, as shown in Fig. 7(b). In the vicinity of the crack tip, particle tracking-based estimation generates strain data very close to the crack tip, but a noticeable void is observed in DIC measurement. The agreement with DIC away from the free boundary confirms the effectiveness of our proposed $\textbf{F}$ estimator and derivative kinematic quantities such as the Eulerian-Almansi strain. The measurements appear to be accurate near free surfaces, demonstrating the significant advantage of the particle tracking-based method in such scenarios.

**Fig. 10:**
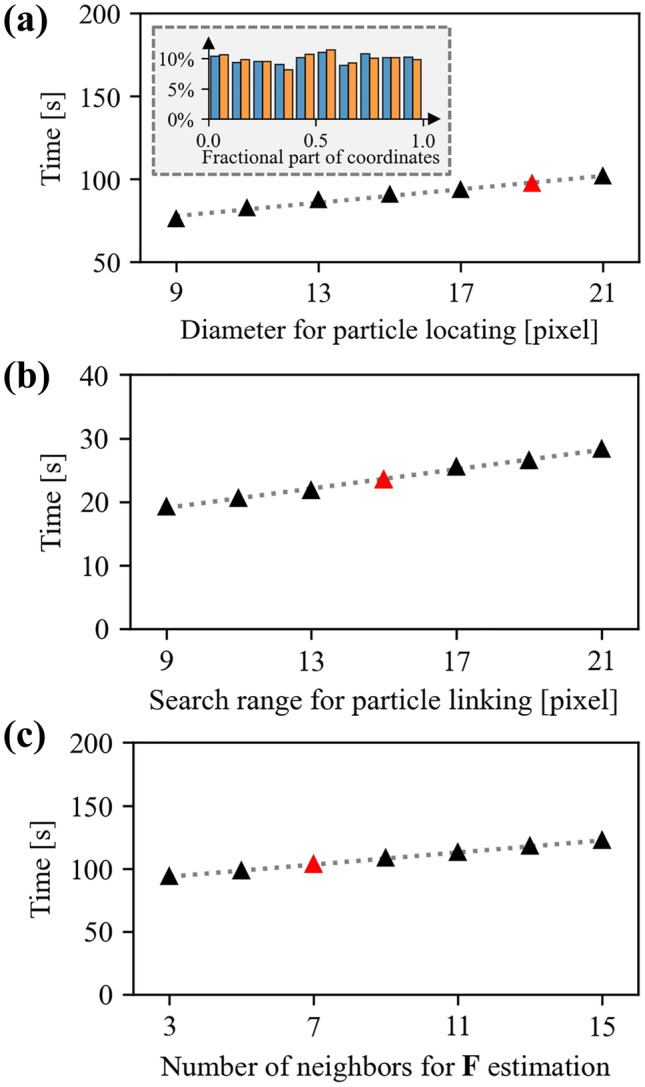
Time consumption for particle tracking and $\textbf{F}$ estimation for a total 251 frames. Red marks indicate the parameters that are used for calculating deformation fields shown in Figs. 7–9. The grey dashed line represents a linear regression of the data as a guide to the eye. (**a**) The time cost for locating particles is plotted as the estimate particle diameter in Trackpy [59]. Inset: the histogram of the fractional part of particles’ coordinates *x* (blue) and *y* (orange) located with a diameter of 19 pixels. As the particles are randomly distributed in the sample, the good uniformity of the distribution confirms the subpixel accuracy of particle locations. (**b**) The time cost for linking particles in consecutive frames as a function of the search range. A search range of 15 pixels is selected for our experiments; this ensures reliable linking of particles near a crack tip as shown in Fig. 1(b). (**c**) The time cost for estimating $\textbf{F}$ as a function of the number of involved neighboring particles. 7 nearest-neighbor particles are used in the estimation of $\textbf{F}$, as this value provides a good compromise between estimation accuracy and spatial resolution of strong gradients

The time consumed for particle tracking, particle linking, and $\textbf{F}$ estimation is evaluated and plotted in Fig. 10, with respect to the indicated parameter. All the computations for the total 251 frames are done in a desktop computer with a 4-core Intel i8-7700K CPU, 64 GB RAM, and a Nvidia Titan Xp GPU.

In general, the particle locating and $\textbf{F}$ estimation are the two most time-consuming steps, significantly exceeding the time required for particle linking. For all three processes, the computational time increases approximately linearly with the primary parameter: the time for locating particles linearly increases with the given particle diameter estimate; the time for particle linking linearly increases with search radius; the time for $\textbf{F}$ estimation is proportional to the number of neighbor particles involved for the estimator. In all calculated deformation fields throughout the manuscript, red-annotated parametric values in Fig. 10 are used; these deformation data are shown in Figs. 7–9. These primary parameter values ensure sub-pixel accuracy of particle locating (inset of Fig. 10(a)), robustness of particle linking, and reliable non-smoothed estimation of local $\textbf{F}$.

Hundreds of seconds are required to calculate the full-field deformation gradient tensor for 251 frames. In contrast to DIC, particle tracking generally requires a higher frame rate, especially in our fracture experiment, where the displacement can be large near the crack tip. To obtain the DIC results shown in Figs. 7 and 9, 26 frames (approximately 1/10 of those used in particle tracking) are calculated with Ncorr [60] using the same hardware, which takes more than a thousand seconds. Increasing the number of frames used would linearly increase the computational time to carry out the DIC. The comparison of computational efficiency between the two methods is primitive; indeed, our implementation of the proposed estimator might not be fully optimized, and furthermore, commercial software for both particle tracking and DIC is likely to improve the tracking/correlation performance. Nevertheless, this comparison indicates that the particle tracking-based method is a competitive alternative to DIC for deformation measurement in terms of computational cost, and its merits exhibited in the fracture experiment demonstrate strong potential for improving kinematic measurements that comprise large deformation or rotation, especially near sample boundaries.

The DIC implementation used in this manuscript is carried out with open-source software, Ncorr [60]. Since the development of this software, DIC has seen significant advances [16, 20, 25, 31], and commercial implementations may be even more capable of addressing some of the free-boundary artifacts identified here. Despite the shortcomings of the DIC implementation we used, we made a significant effort to optimize the accessible DIC parameters, and thus the comparison is informative as both particle tracking and DIC software employed are state-of-the-art open source versions.

### 3D Uniaxial Tensile Test

We further apply particle tracking to 3D displacement measurement in a uni-axially stretched hydrogel sample, and extend our proposed estimator to 3D in order to estimate $\textbf{F}$ and strain fields. The sample is incrementally loaded with uni-axial stretch using the same experimental setup shown in Fig. 5(a), and a 100 µm-thick volume is recorded in image stacks by varying the relative position of the objective with a step size of 2 µm.

**Fig. 11:**
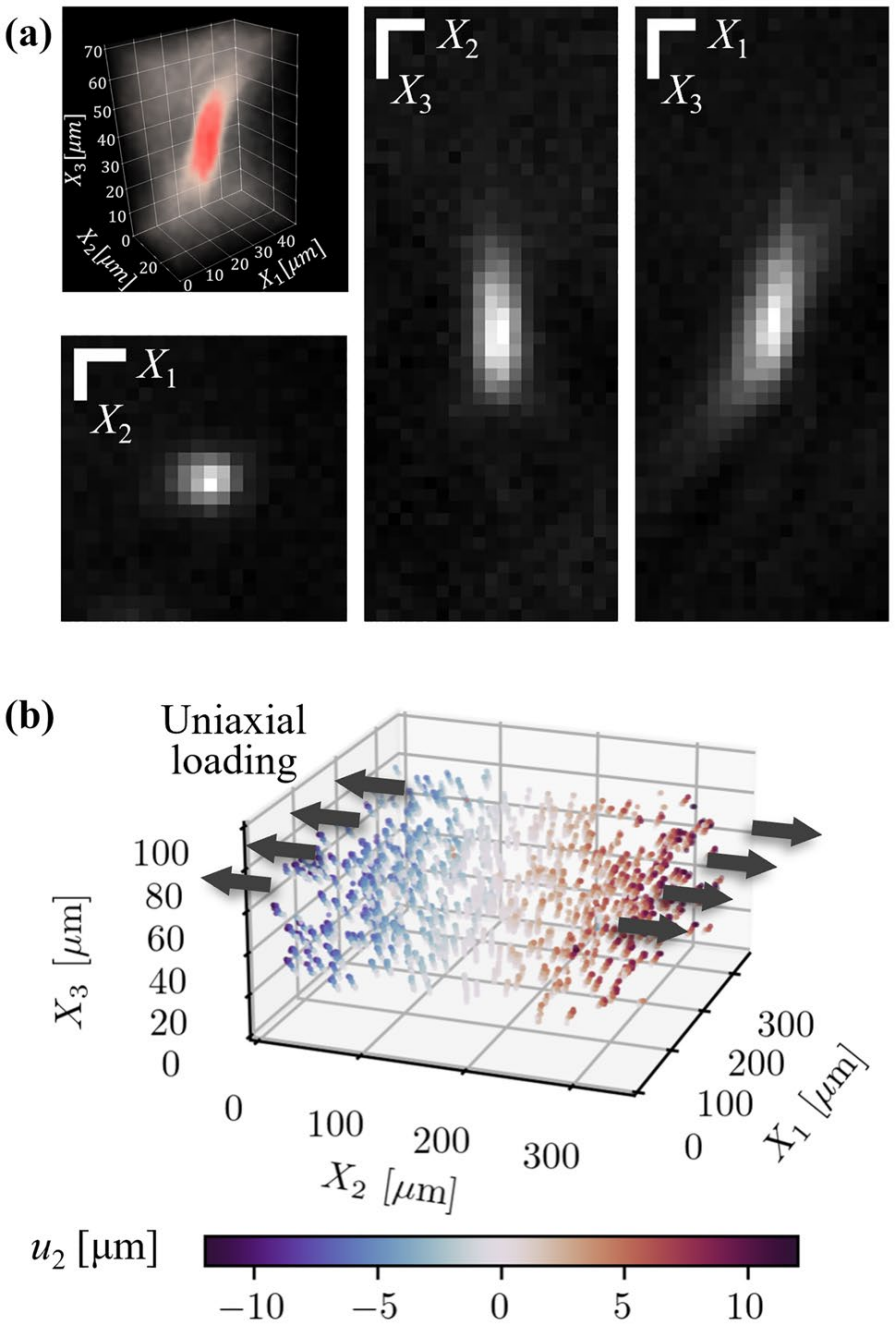
3D particle tracking in a uniaxial tensile test. A hydrogel sample is uniaxially stretched by a total of 15 incremental loading steps, using the experimental setup shown in Fig. 5(a). (**a**) A 3D view of the light scattered by a single particle, and imaged in the image stack. The image intensity is encoded by color. The cross-sections of the particle through its center in the $X_1X_2$, $X_2X_3$, and $X_3X_1$ planes as indicated. Scale bars in the upper left corners represent 2 µm in the $X_1$ and $X_2$ direction, and 10 µm in the $X_3$ direction. (**b**) 3D Particle trajectories color-coded by the value of the displacement component $\hat{u}_2$. Note that the aspect ratio of the axes is not equal, for the sake of clarity of particle trajectories; thus, the out-of-plane displacement $u_3$ is exaggerated

The particles are randomly distributed in the recorded volumetric image. Despite the sphericity of the particles, they appear as elongated ellipses in the images, with the major axis oriented parallel to the $X_3$ axis, as can be seen in the magnified volumetric image of the scattered intensity field recorded for a single particle in the top left inset of Fig. 11(a). The ellipsoidal particle shape is also rendered in cross-sections through the center of the particle, as shown in Fig. 11(a). The particle appears to be an isotropic Gaussian blob in the $X_1X_2$ plane, but appears greatly elongated along $X_3$. The elongated particle shape occurs as a consequence of the out-of-focus light, which can be eliminated by confocal microscopy. Also, due to the imperfect illumination and as a result the asymmetric point spread function (PSF), a slight, but still observable, tilt of the particle appears in the $X_3X_1$ cross-section.

Despite the deficiency of the imaging system’s illumination scheme, particles are robustly located and linked in 3D by Trackpy [59] for a total of 16 volumes. Particle trajectories are plotted in 3D in Fig. 11(b), and color-coded according to the displacement component, $u_2$. As can be seen from the trajectories, the particles displace predominantly along the loading direction, $X_2$, with an apparent displacement gradient; displacement along $X_3$ is also substantial; however, this displacement is exaggerated in the trajectory plot due to the aspect ratio of the axes. The displacement fields, calculated in the last frame, are then used to estimate $\textbf{F}$ with our proposed estimator given by equation (2), at each particle location with 15 neighboring particles. The Green-Lagrange finite strain tensor is further inferred from $\hat{\textbf{F}}$, and the uniaxial strain is measured at 0.069, with a small standard deviation of $4.1\%$ over all measurements. This experiment, by extending particle tracking from 2D to 3D while maintaining the accuracy and robustness, confirms the effectiveness of our proposed estimator in the evaluation of 3D deformation gradients.

## Discussion

Although the estimator given by equation (2) can be shown to be the MMSE with respect to $\varepsilon _\text {M}$, it must be noted that this does give any guarantee on $\varepsilon _\text {FD}$. Indeed, higher-order methods exist and can reduce FD error. However, they also require meeting a set of constraints which conflict with $\varepsilon _\text {M}$’s optimality. Depending on the displacement field and measurement noise, higher-order methods may or may not improve accuracy.

The theoretical description of expected error in equation (4) gives insights into the variation of the error. Not only does it enable a computation of expected measurement error in physical experiments, but it highlights the importance of several parameters, in particular, the matrix $\textbf{A}$ defined in equation (4) which contains information on the geometry of the problem. The inverse of $\textbf{A}$ in the estimation of deformation gradients can be used to determine an upper-bound of the error, which depends linearly on $\frac{1}{\lambda _\text {min}(\textbf{A})}$. Although normalized by $\frac{1}{k}$, its eigenvalues do vary with *k*. For instance, to ensure invertibility, *k* must be larger than the number of spatial dimensions. Nevertheless, even when $\textbf{A}$ is invertible, if *k* is relatively small, neighbors could be closely aligned with high probability, leading to poor SNR of the displacement variation along certain directions. The noise in the estimated deformation gradient would be amplified along these directions, as a consequence of the small minimal eigenvalue of $\textbf{A}$, $\lambda _\text {min}(\textbf{A})$. However, as *k* increases, neighbors are less and less likely to be aligned; as a consequence, richer measurements of displacement variation are more probable, leading to a better eigenvalue distribution of $\textbf{A}$ and lower estimator variance. For $k\rightarrow \infty$, $\textbf{A}$ and its inverse converge almost surely to a multiple of the identity matrix. Consequences of the properties of $\textbf{A}$ are visible in the numerical experiments reported in Fig. 3(b), where standard deviation decreases as *k* increases. Error also decreases due to an increasing smallest eigenvalue before approaching an asymptotic limit.

The proposed estimator is developed based on the particle tracking method. Both the particle tracking and DIC methods can realize non-contact, full-field displacement measurement with similar computational cost, and can be extended to 3D measurements, depending on the imaging system. Indeed, DIC has become more popular than particle tracking in experimental solid mechanics, thanks to its gridded data format. With the proposed estimator, non-gridded data is no longer a problem for estimating $\textbf{F}$, and particle tracking demonstrates several distinctive advantages in deformation measurement. First, unlike DIC, particle tracking does not require dense speckle patterns, and thus, it can have a flexible selection of particle density, which is beneficial for applications where dense particles are not desired, e.g., in biological systems. Second, particles are located in individual images and then linked across images in order to obtain trajectories. As a consequence, the accuracy of particle tracking is independent of the extent of deformation, circumventing the decorrelation or cumulative errors that arise when DIC is applied in situations of large deformation [26, 27]. Moreover, particles offer the advantage that they can be fully embedded, and thus when subject to very large deformation, they do not break their attachment to the sample [63]. Third and most poignantly, particle tracking is effective extremely close to free surfaces and under large deformation and/or rotation, as demonstrated in the fracture experiment. The method’s robustness can be guaranteed by a sufficient frame rate. In circumstances where a high frame rate cannot be achieved, a hybrid DIC-aided tracking method is suggested [64], where sparse DIC calculations can be used to provide reliable prediction for particle linking.

More attention is advocated for the deformation gradient tensor. In the wide application of experimental methods to the measurement of deformation, strain tensors are used extensively; however, deformation cannot be fully described by the strain tensors. For example, in the previously described fracture experiment, material rotation is measured to be more than $30 \deg$ near the crack tip by polar decomposition of $\textbf{F}$ [62], which otherwise cannot be extracted from finite strain tensors or from the linearized strain tensor. Furthermore, novel constitutive models, e.g., hyperelastic material model [65, 66], require the deformation gradient tensor to correctly describe the nonlinear material behavior. Finally, several analytical tools in solid mechanics, e.g. the *J*-integral [67, 68], also use kinematic information exclusively available in $\textbf{F}$. Our proposed estimator, from the experimental perspective, enables the application of the theoretically-developed novel material models and mechanics tools by accurately estimating $\textbf{F}$.

## Conclusion

In this manuscript, a technique for estimating the deformation gradient is developed and analyzed based on a set of randomly dispersed displacements measured by particle tracking. The deformation gradient estimator given by equation (2) is derived in detail, and its expected error is decomposed and expanded into measurement error $\varepsilon _\text {M}$ and FD error $\varepsilon _\text {FD}$. Based on the standard deviation of displacement measurements, $\varepsilon _\text {M}$ can be computed for estimation of error in experiments. Computation of $\varepsilon _\text {FD}$ is impossible without knowledge of second order derivatives of displacement. This estimator is subjected to a variety of tests using simulated data, and then employed in the analysis of physical experiments. In simulations, FD error is seen to follow the rate $\mathcal {O}(R)$. Furthermore, it decreases as the number of nearest neighbors grows, *k*, before reaching a nonzero asymptotic limit. As for measurement error, numerical simulations boast a $\mathcal {O}(\frac{\sigma }{R \sqrt{k}})$, corroborating that measurement noise is filtered when the number of neighbors increases. In physical experiments, the accuracy of the estimator is verified in both 2D and 3D, and its effectiveness in measuring complicated deformation such as occurs adjacent to free boundaries is confirmed. Our proposed estimator, based on particle tracking, is expected to open a door for future material tests and experimental mechanics study, particularly in soft materials that routinely undergo large deformation or substantial rotation, especially near their boundaries.

## Figures and Tables

**Fig. 12:**
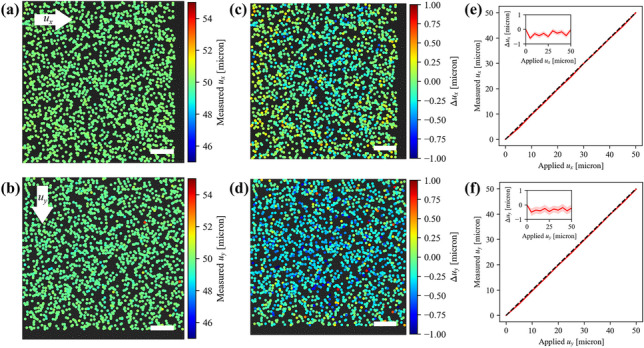
Experimental results of translation tests in the *x*- and *y*-directions. (**a**) and (**b**) Displacement fields tracked at particle locations after 50 µm translation in the *x*- and *y*-direction, respectively. The deviations of these measured displacement fields to the prescribed 50 µm displacement are shown in (**c**) and (**d**). The scale bars are 100 µm. (**e**) and (**f**) The mean measured displacement in the *x*- and *y*-directions after each translation increment of 5 µm. Dark dashed lines with a slope of 1 are plotted as a guide to the eye. Inset: the mean deviation of the measured displacement from the applied displacement is calculated for all particles. The standard deviation is indicated by the shaded region

## Data Availability

The data that support the findings of this study are openly available via Zenodo at https://zenodo.org/record/8169372, with 10.5281/zenodo.4410128.

## Appendix 1 Estimator Error

Following “Estimator Derivation and Theory of Estimator Error” section, the aim is to estimate the deformation gradient of a center particle 0, by measuring displacements $\hat{\textbf{u}}_0,\dots ,\hat{\textbf{u}}_k$ of it and its *k*-nearest neighbors, randomly dispersed at positions $\textbf{r}_0,\dots ,\textbf{r}_k$. Each of the $k+1$ particles lie in the search-ball $\Omega$ of radius *R*, centered at $\textbf{r}_0$.

Measurements are assumed to be independent Gaussian variables such that $\epsilon _{n,i} = \hat{u}_{n,i} - u_i(\textbf{r}_n) \sim \mathcal {N}(0,\frac{\sigma ^2}{3})$. Emphasis is put on obtaining an exact expression of estimator (2)’s mean square error (MSE).

To obtain this expression, the difference $\nabla u_i(\textbf{r}_0) - \hat{\nabla } u_i(\textbf{r}_0)$ is derived for each component *i* of the displacement field, by including the remainder of the Taylor expansion in equation (1). Then, the error is computed by expanding the Frobenius norm

10 $$\begin{aligned} \begin{aligned} \Vert \mathbf {F(r}_0) - \hat{\textbf{F}(r}_0)\Vert _F^2&= \sum _{i=1}^3 \sum _{j=1}^3(F_{ij}(\textbf{r}_0) - \hat{F}_{ij}(\textbf{r}_0))^2 \\&= \sum _{i=1}^3 \Vert \nabla u_i(\textbf{r}_0) - \hat{\nabla } u_i(\textbf{r}_0)\Vert ^2 \end{aligned} \end{aligned}$$

Beginning with the derivation of the difference $\nabla u_i(\textbf{r}_0) - \hat{\nabla } u_i(\textbf{r}_0)$, a Taylor expansion including remainder is expanded for displacement component *i*. $\varvec{\xi }_n^i$ is a point along the line ranging from $\textbf{r}_0$ to $\textbf{r}_n$. This point is different for each component *i* of displacement. The Taylor expansion holds for all neighbors $1,\dots ,k$ provided that $u_i$ is twice continuously differentiable everywhere in the search ball $\Omega$. In experiments, as long as neighbors are not included across cracks or sharp lobes, this condition should be satisfied.

11 $$\begin{aligned} u_i(\textbf{r}_n) = u_i(\textbf{r}_0) + \textbf{d}_n^\textrm{T} \nabla u_i(\textbf{r}_0) + \frac{1}{2} \textbf{d}_n^\textrm{T} \nabla ^2 u_i(\varvec{\xi }_n^i) \textbf{d}_n \end{aligned}$$

Each of the $n=1,\dots ,k$ equations are multiplied by $\textbf{d}_n$. Then, they are summed together.

$$\begin{aligned} \begin{aligned} \sum _{n=1}^k \textbf{d}_n \textbf{d}_n^\textrm{T} \nabla u_i(\textbf{r}_0) =&\ \sum _{n=1}^k (u_i(\textbf{r}_n) - u_i(\textbf{r}_0))\textbf{d}_n \\&- \frac{1}{2} \sum _{n=1}^k \textbf{d}_n^\textrm{T} \nabla ^2 u_i(\varvec{\xi }_n^i) \textbf{d}_n \, \textbf{d}_n \end{aligned} \end{aligned}$$

Multiplication by the inverse of $\sum _{n=1}^k \textbf{d}_n \textbf{d}_n^\textrm{T}$ yields an expression of row *i* of the deformation gradient $\textbf{f}_i$. It can be subtracted from the estimator given by equation (2)’s $i^\text {th}$ row, $\hat{\textbf{f}}_i$,

$$\begin{aligned} \begin{aligned} \hat{\textbf{f}}_i - \textbf{f}_i &= \left(\sum _{n=1}^k \textbf{d}_n \textbf{d}_n^\textrm{T})^{-1} \sum _{n=1}^k (\epsilon _{n,i} - \epsilon _{0,i}\right) \textbf{d}_n \\&\quad+ \frac{1}{2} {\left(\sum _{n=1}^k \textbf{d}_n \textbf{d}_n^\textrm{T}\right)^{-1}} \sum _{n=1}^k \textbf{d}_n^\textrm{T} \nabla ^2 u_i(\varvec{\xi }_n^i) \textbf{d}_n \, \textbf{d}_n \text {.} \end{aligned} \end{aligned}$$

$\textbf{A} = \frac{1}{R^2 k} \sum _{n=1}^k \textbf{d}_n \textbf{d}_n^\textrm{T}$ is defined. Furthermore,

12 $$\begin{aligned} \begin{aligned} \textbf{X}_i&= \frac{1}{R^2 k} \textbf{A}^{-1} \sum _{n=1}^k (\epsilon _{n,i} - \epsilon _{0,i}) \textbf{d}_n \\ \textbf{Y}_i&= \frac{1}{R^2 k} \textbf{A}^{-1} \frac{1}{2} \sum _{n=1}^k \textbf{d}_n^\textrm{T} \nabla ^2 u_i(\varvec{\xi }^i_n) \textbf{d}_n \, \textbf{d}_n \end{aligned} \end{aligned}$$

The expectation conditioned on $\{\textbf{d}_n\}_{n=1}^k$ of the square error of the estimator is taken. Recognizing that $\mathbb {E}[\textbf{X}_i \mid \{\textbf{d}_n\}^k_{n=1}] = 0$ and that $\textbf{Y}_i \mid \{\textbf{d}_n\}_{n=1}^k$ is constant

13 $$\begin{aligned} \mathbb {E} [\Vert \hat{\textbf{f}}_i - \textbf{f}_i \Vert ^2 \mid \{\textbf{d}_n\}_{n=1}^k ] = \mathbb {E}[\Vert \textbf{X}_i\Vert ^2 \mid \{\textbf{d}_n\}_{n=1}^k ] + \Vert \textbf{Y}_i\Vert ^2 \end{aligned}$$

First, a few steps are taken to expand the conditional expectation of $\Vert \textbf{X}_i\Vert ^2$.

14 $$\begin{aligned} &{\mathbb {E}[\Vert \textbf{X}_i\Vert ^2 \mid \{\textbf{d}_n\}_{n=1}^k ] = \frac{1}{R^4 k^2} \sum _{m=1}^k\sum _{n=1}^k \textbf{d}_m^\textrm{T} \textbf{A}^{-\textrm{T}}}\nonumber \\ &{\textbf{A}^{-1} \textbf{d}_n \mathbb {E}[(\epsilon _{m,i}-\epsilon _{m,0})(\epsilon _{n,i}-\epsilon _{n,0})]} \end{aligned}$$

Mutual independence of the various $\epsilon _{n,i}$ implies that $\mathbb {E}[\epsilon _{m,i}\epsilon _{n,i}] = \frac{\sigma ^2}{3}\delta _{nm}$ where $\delta _{nm}$ is the Kronecker delta. Equation (14) becomes

15 $$\begin{aligned} \begin{aligned}\mathbb {E}[\Vert \textbf{X}_i\Vert ^2 \mid \{\textbf{d}_n\}_{n=1}^k ]&= \frac{1}{R^4 k^2} \sum _{m=1}^k\sum _{n=1}^k \textbf{d}_m^\textrm{T} \textbf{A}^{-\textrm{T}} \textbf{A}^{-1} \textbf{d}_n \frac{\sigma ^2}{3}(\delta _{nm} + 1) \\&= \frac{\sigma ^2}{3 R^2 k} \{ \frac{1}{k}\sum _{n=1}^k \frac{\textbf{d}_n^\textrm{T}}{R} \textbf{A}^{-\textrm{T}}\textbf{A}^{-1} \frac{\textbf{d}_n}{R} \\&\quad + k(\textbf{A}^{-1} \frac{1}{k}\sum _{m=1}^k \frac{{\textbf {d}}_m}{R} )^\textrm{T} \textbf{A}^{-1} \frac{1}{k}\sum _{n=1}^k \frac{{\textbf {d}}_n}{R} \} \end{aligned} \end{aligned}$$

Exploiting the fact that

16 $$\begin{aligned} {\Vert \textbf{M}\textbf{v}\Vert ^2 = \text {tr} (\textbf{v}\textbf{v}^\textrm{T} \textbf{M}^\textrm{T}\textbf{M})} \end{aligned}$$

for any matrix ${\textbf {M}}$ and vector ${\textbf {v}}$ of compatible sizes, equation (15) can be recast into

17 $$\begin{aligned} \begin{aligned}&\mathbb {E}[\Vert \textbf{X}_i\Vert ^2 \mid \{\textbf{d}_n\}_{n=1}^k ] \\&= \frac{\sigma ^2}{3 R^2 k} \{ \text {tr}(\frac{1}{k}\sum _{n=1}^k \frac{\textbf{d}_n}{R} \frac{\textbf{d}_n^\textrm{T}}{R} \textbf{A}^{-\textrm{T}}\textbf{A}^{-1} ) \\&\,\,\,\,\,\,\, + k \Vert \textbf{A}^{-1} \frac{1}{k}\sum _{n=1}^k \frac{{\textbf {d}}_n}{R}\Vert ^2 \} \\&= \frac{\sigma ^2}{3 R^2 k} \{ \text {tr}(\textbf{A} \textbf{A}^{-\textrm{T}}\textbf{A}^{-1} ) \\&\,\,\,\,\,\,\, + k \Vert \textbf{A}^{-1} \frac{1}{k}\sum _{n=1}^k \frac{{\textbf {d}}_n}{R}\Vert ^2 \} \end{aligned} \end{aligned}$$

Finally, by symmetry of $\textbf{A}$,

18 $$\begin{aligned} {\text {tr}(\textbf{A} \textbf{A}^{-\textrm{T}} \textbf{A}^{-1}) = \text {tr}(\textbf{A}^{-1})} \end{aligned}$$

and the conditional expectation of $\Vert \textbf{X}_i\Vert ^2$ is

19 $$\begin{aligned} \mathbb {E}[\Vert \textbf{X}_i\Vert ^2 \mid \{\textbf{d}_n\}_{n=1}^k ] &= \frac{\sigma ^2}{3 R^2 k}(\text {tr}(\textbf{A}^{-1}) \\ & \quad+ k \Vert \textbf{A}^{-1} \frac{1}{k}\sum _{n=1}^k \frac{\textbf{d}_n}{R} \Vert ^2) \end{aligned}$$

Then, taking the norm of $\textbf{Y}_i$ yields

20 $$\begin{aligned} \Vert \textbf{Y}_i\Vert ^2 = \frac{R^2}{4} \Vert \frac{1}{k} \sum _{n=1}^k \frac{\textbf{d}_n^\textrm{T}}{R} \nabla ^2 u_i(\varvec{\xi }^i_n) \frac{\textbf{d}_n}{R} \, \textbf{A}^{-1} \frac{\textbf{d}_n}{R} \Vert ^2 \end{aligned}$$

Measurement and Finite Difference (FD) errors, $\varepsilon _\text {M}$ and $\varepsilon _\text {FD}$ are defined.

21 $$\begin{aligned} \begin{aligned} \varepsilon _\text {M}&= \sqrt{\sum _{i=1}^3 \mathbb {E}[\Vert \textbf{X}_i\Vert ^2 \mid \{\textbf{d}_n\}_{n=1}^k ]}\\&= \frac{\sigma }{R \sqrt{k}}\sqrt{\text {tr}(\textbf{A}^{-1}) + k \Vert \textbf{A}^{-1} \frac{1}{k}\sum _{n=1}^k \frac{\textbf{d}_n}{R} \Vert ^2} \end{aligned} \end{aligned}$$

22 $$\begin{aligned} \begin{aligned} \varepsilon _\text {FD}&= \sqrt{\sum _{i=1}^3 \Vert \textbf{Y}_i\Vert ^2}\\&= \frac{R}{2} \sqrt{\sum _{i=1}^3 \Vert \frac{1}{k} \sum _{n=1}^k \frac{\textbf{d}_n^\textrm{T}}{R} \nabla ^2 u_i(\varvec{\xi }^i_n) \frac{\textbf{d}_n}{R} \, \textbf{A}^{-1} \frac{\textbf{d}_n}{R} \Vert ^2} \end{aligned} \end{aligned}$$

Finally, an expression of the MSE of estimator (2) can be obtained from the Frobenius norm.

23 $$\begin{aligned} \begin{aligned} \mathbb {E}[\Vert \mathbf {F(r}_0) -&\hat{\textbf{F}(r}_0)\Vert _F^2 \mid \{\textbf{d}_n\}_{n=1}^k ] \\&= \sum _{i=1}^3 \mathbb {E}[\Vert \textbf{X}_i\Vert ^2 \mid \{\textbf{d}_n\}_{n=1}^k ] + \sum _{i=1}^3 \Vert \textbf{Y}_i\Vert ^2 \\&= \varepsilon _\text {M}^2 + \varepsilon _\text {FD}^2 \end{aligned} \end{aligned}$$

## Appendix 2 Translation Test

Translation experiments are carried out in the *x*- and *y*-directions with the experimental setup shown in Fig. 5(a). For each direction, the sample is translated by a precise translation stage (Marzhauser-Wetzlar, Tango controller) for 10 steps with a step size of 5 µm (scale: $1 \text {pixel} = 0.43 \mu$m). Images in the initial state and each translated state are recorded, and processed with the procedures and parameters that have been used in the experiments described in the main text. Displacement fields in final states, i.e., after 50 µm translation in the *x*- or *y*-direction, are calculated based on particle tracking, and shown in Fig. 12(a) and (b), respectively. The deviations of the measured displacements from the applied ones are plotted in Fig. 12(c) and (d), for *x*- and *y*-translation, respectively. It is seen that the particles tracked in the *y*-translation test show a larger deviation than those in the *x*-translation test. At each translation step, the mean value of the displacements measured for all the particles are calculated and plotted in Fig. 12(e) and (f), for translation along *x* and *y*, respectively. The average measured displacements boast agreement with the prescribed value, and the deviations shown in the insets also confirm submicron accuracy. Despite the larger standard deviation in the *y*-translation test than in the *x*-translation test, the average standard deviation for 10 translation steps in both tests are within half a micron, i.e., 0.15 micron (0.35 pixel) for *x*-translation and 0.24 micron (0.56 pixel) for *y*-translation. From this base-line noise measurement, we set the noise level at 0.455 pixel ($\frac{0.56+0.35}{2}$) for the theoretical calculations in the main text.
